# Automatic compensation enhances the orientation perception in chronic astigmatism

**DOI:** 10.1038/s41598-022-07788-y

**Published:** 2022-03-08

**Authors:** Sangkyu Son, Won Mok Shim, Hyungoo Kang, Joonyeol Lee

**Affiliations:** 1 Center for Neuroscience Imaging Research, Institute for Basic Science (IBS), Suwon, 16419 Republic of Korea; 2 Department of Biomedical Engineering, Sungkyunkwan University, 2066 Seobu-ro, Jangan-gu, Suwon-si, Gyeonggi-do 16419 Republic of Korea; 3 Department of Optometry, Catholic Kwandong University, 24, Beomil-ro 579beon-gil, Gangneung-si, Gangwon-do 25601 Republic of Korea; 4 Department of Intelligent Precision Healthcare Convergence, Sungkyunkwan University, Suwon, 16419 Republic of Korea

**Keywords:** Psychophysics, Sensory processing, Bayesian inference, Human behaviour, Perception, Neural decoding

## Abstract

Astigmatism is a prevalent optical problem in which two or more focal points blur the retinal image at a particular meridian. Although many features of astigmatic vision, including orientation perception, are impaired at the retinal image level, the visual system appears to partly restore perceptual impairment after an extended period of astigmatism. However, the mechanism of orientation perception restoration in chronic astigmatism has not yet been clarified. We investigated the notable reduction of perceptual error in chronic astigmatism by comparing the orientation perception of a chronic astigmatism group with the perception of a normal-vision group, in which astigmatism was transiently induced. We found that orientation perception in the chronic group was more accurate than in the normal vision group. Interestingly, the reduction of perceptual errors was automatic; it remained even after the optical refractive errors were fully corrected, and the orientation perception was much more stable across different orientations, despite the uneven noise levels of the retinal images across meridians. We provide here a mechanistic explanation for how the compensation of astigmatic orientation perception occurred, using neural adaptation to the biased distribution of orientations.

## Introduction

Astigmatism is a common visual problem in which light rays focus unevenly on two or more focal lines, resulting in a retinal image that is blurred at one meridian. Most people have some degree of astigmatism^1,2^; however, uncorrected astigmatism significantly impairs the fundamental aspects of vision, such as visual acuity^3,4^ and contrast sensitivity^5^. Thus, ophthalmologists always include an indicator of astigmatic aberrations in eyeglass prescriptions.

**Figure 1:**
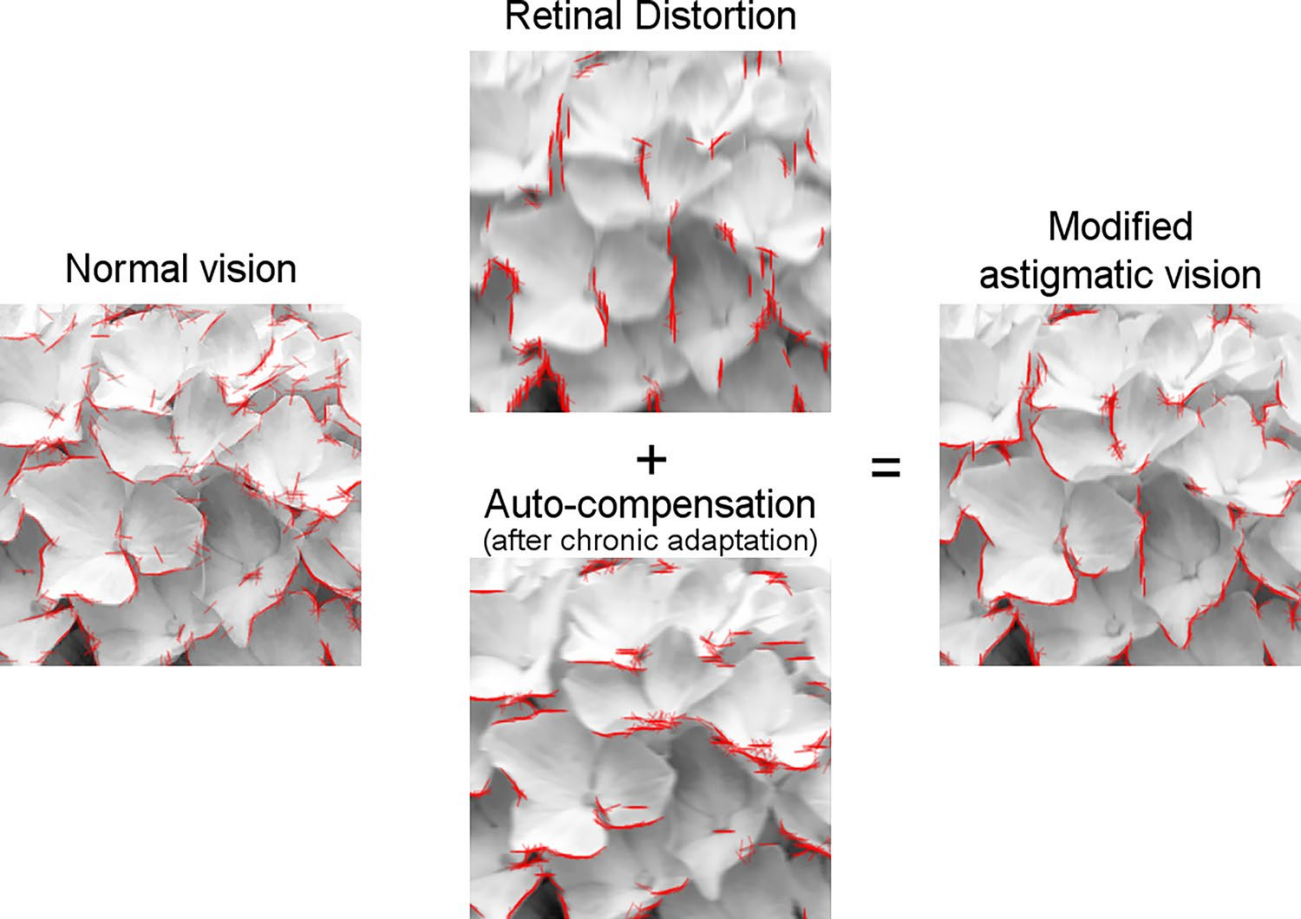
Simulation of the compensatory effect on chronic astigmatism when an image of a hydrangea is presented. The effect of the astigmatic blur and the automatic compensation were simulated for visualization purposes, according to the mechanisms of the adaptation model described in the Results and Methods sections. The edges of each image were detected with the Sobel operator (red). The edges are intact in the image of normal vision but severely biased vertically in the astigmatic retinal image. After being counterbalanced by the inversely biased edges of the automatic compensation, the vision with chronic astigmatism partly restores the original edges.

Owing to the meridian-specific characteristics of astigmatism, it also impairs orientation perception by distorting the retinal images and resultant orientation representations in the brain^6^. Because the optical blur is maximum at the orientation of the astigmatic axis, the edges and lines of the visual scenes are systematically distorted. As a result, the orientation information is biased away from the astigmatic axis and heavily concentrated around the orientation that is orthogonal to the astigmatic axis. The image in the upper panel of Fig. 1 shows an example simulation of the resulting astigmatic retinal distortion; when we appreciate a natural scene of beautiful hydrangeas, the outlines of the petals appear to be cut off, and even the remaining edges appear oddly skewed. Fortunately, our visual system somehow partly reduces the effect of the optical blur^7^, allowing us to see the natural continuous outlines of petals (Fig. 1, right panel).

Previous studies have reported several possible ways to reduce the perceptual errors induced by astigmatism. Some studies have shown that adapting to the astigmatic blur can compensate for the perceptual errors in non-astigmatic^8,9^ or chronic astigmatic eyes^10^. Prior knowledge can also reduce perceptual errors because it is known that long-term exposure to certain statistics of natural visual scenes can reprogram the visual system for efficient information encoding^11–13^.

Despite the proposed possibilities, we still lack a mechanistic understanding of the neural processes underlying orientation identification correction and how perception accuracy is improved in astigmatism. To address these issues, we compared the perceptual errors and orientation-specific optical blurs of individuals with chronic astigmatism and those with normal vision, wherein we transiently induced astigmatism. We found that distortion of orientation perception was compensated for much more in individuals with chronic astigmatism than in those with experimentally induced astigmatism. The compensation was automatic and remained even after the complete correction of the optical aberration. The variability of the perceptual errors across trials remained constant regardless of stimulus orientations, even though the meridian-specific feature of astigmatism causes different sizes of optical noise across different orientations, thus indicating the automatic property of compensation. We further suggested a mechanistic model that can explain the reduction in orientation distortion in chronic astigmatism, by assuming a simple neural adaptation to specific orientations.

## Results

We presented multiple Gabor stimuli with different orientations to eyes with chronic astigmatism (chronic group) and to normal-vision eyes with a cylindrical lens to induce astigmatism (control group). In the chronic group, datasets from 47 eyes (from 27 participants) were collected with their own cylindrical refractive errors (Supplementary Fig. S1, upper left panel), which ranged from − 0.25° to − 4.00° diopter (D) with a mean of − 1.73 (Table 1). In the control group, a cylindrical lens varying from 1.00 to 4.00 D was put to 10 dominant eyes (from 10 participants) in random order to simulate the various amounts of astigmatism (Supplementary Fig. S1, lower left panel). The participants reported the perceived mean orientations of the Gabor stimuli by rotating a white bar using a mouse at the end of each trial (Fig. 2B).

**Table 1:** The measured refractive errors of the chronic group.

#	Sex	Age	Right eye refractive error					Left eye refractive error				
			Naked			Daily uncorrected		Naked			Daily uncorrected	
			Spherical (diopter)	Cylindrical (diopter)	Axis (degree)	Spherical (diopter)	Cylindrical (diopter)	Spherical (diopter)	Cylindrical (diopter)	Axis (degree)	Spherical (diopter)	Cylindrical (diopter)
01	Male	19	− 6.50	− 0.75	10	− 0.50	− 0.50	− 5.75	− 1.25	160	− 0.25	− 0.75
02	Male	23	− 4.25	− 2.75	0	− 0.25	0	− 5.00	− 3.00	175	− 0.50	+ 0.25
03	Male	19	− 17.00	− 1.00	175	− 3.00	− 1.00	−				
04	Male	19	−					− 3.25	− 1.25	0	− 0.25	− 0.50
05	Female	19	− 7.25	− 2.25	0	+ 0.25	+ 0.25	− 6.75	− 1.00	170	0	0
06	Male	32	− 9.75	− 1.25	5	0	− 0.50	− 10.5	− 1.00	175	− 0.75	− 0.25
07	Male	20	− 3.00	− 0.25	15	0	+ 0.25	− 2.00	− 1.50	165	+ 0.25	− 0.25
08	Male	19	− 4.50	− 2.75	170	0	− 0.75	− 3.75	− 2.25	0	− 0.50	− 0.25
09	Female	20	− 5.25	− 1.00	165	− 0.25	− 0.25	− 5.00	− 1.50	180	− 0.00	− 0.50
10	Male	19	− 5.00	− 2.00	5	0	0	− 4.75	− 1.75	165	− 0.00	0
11	Male	19	− 4.25	− 1.75	5	− 0.50	0	− 4.25	− 1.75	175	− 0.75	− 0.25
12	Male	23	− 5.50	− 2.75	0	0	− 0.75	− 6.00	− 3.25	170	− 0.25	− 0.25
13	Female	19	− 5.00	− 1.75	25	0	− 0.50	− 5.00	− 1.50	165	− 0.00	− 0.25
14	Female	19	–					− 7.25	− 2.75	170	+ 0.25	− 1.00
15	Female	18	− 10.25	− 3.25	0	− 1.75	− 0.25	− 6.50	− 3.75	0	− 0.50	− 0.75
16	Male	22	− 3.75	− 1.00	0	− 0.25	0	− 4.00	− 1.00	0	− 0.25	0
17	Male	19	− 5.50	− 0.75	5	− 1.25	− 0.25	− 3.75	− 1.25	165	− 0.50	− 0.50
18	Female	19	− 2.75	− 0.75	5	− 0.25	0	− 3.00	− 0.75	10	+ 0.25	− 0.75
19	Male	19	− 10.25	− 2.25	170	− 1.25	− 0.25	− 4.50	− 2.00	10	+ 0.50	− 0.25
20	Male	19	− 0.75	− 3.50	0	0	0	−				
21	Male	22	− 6.00	− 2.50	170	− 0.50	− 0.75	− 6.75	− 1.50	170	− 0.75	− 0.50
22	Female	20	− 1.00	− 0.75	170	− 0.25	− 0.75	0	− 2.50	175	0	− 0.50
23	Female	19	–					− 3.50	− 1.00	160	0	− 1.00
24	Male	26	− 4.25	− 3.75	0	0	0	–				
25	Female	19	− 2.75	− 0.75	5	0	− 0.25	− 5.25	− 1.00	170	− 0.50	0
26	Female	20	− 4.25	− 1.00	170	+ 0.75	− 1.00	−				
27	Female	19	− 5.00	− 1.25	70	0	− 0.75	− 5.25	− 1.00	95	− 0.50	+ 0.50

**Figure 2:**
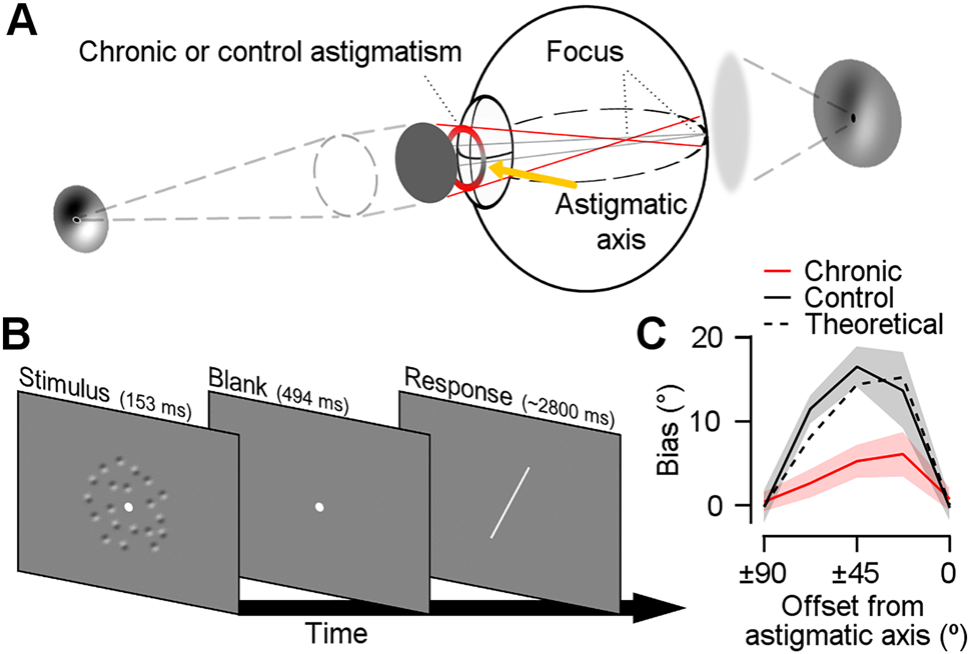
(**A**) Participants viewed a Gabor stimulus either with their own chronic refractive error (chronic group) or experimentally-induced astigmatic refractive error (control group). In both cases, a refractive power on the orthogonal axis is higher than on the astigmatic axis (yellow arrow). The circle in front of the eye indicates the refractive powers of each meridian (the higher refractive power is in red). This causes light rays from the Gabor stimulus to refract more at the orthogonal axis (red lines) than at the astigmatic axis (gray lines), shaping elliptical blur. As a result, the orientation of the Gabor stimulus in the retinal image tilts away from the astigmatic axis (horizontal). We controlled the experiment duration to prevent the participants from adapting to the optical environments. (**B**) A schematic of the orientation adjustment task. Randomly tilted Gabor stimuli were briefly presented at the center. After the post-stimulus blank, participants reported the perceived mean orientation by rotating the orientation bar. (**C**) The perceptual biases of the chronic group (red line), control group (solid black line), and prediction of the theoretical model (dashed black line) were plotted as a function of offset from the astigmatic axis. The shaded areas indicate ± 2 standard error of the mean (SEM).

### Chronic exposure to astigmatism compensates for biases induced by optical blurring in orientation perception

Although the refractive errors in optical organs caused similar orientation distortions in both the chronic and control groups, the biases in the perceived mean orientation were different among the groups (Fig. 2C; two-way analysis of variance (ANOVA), *F*_*group* (1,275)_ = 29.799, *F*_*offset* (4,275)_ = 23.539, *F*_*interaction* (4,275)_ = 7.074, *p*_s_ < 0.001). The difference came primarily from the responses when the stimulus was tilted ± 67.5° and ± 45° away from the astigmatic axis (± 67.5°: *t*_(55)_ = − 4.568, ± 45°: *t*_(55)_ = − 5.015, *p* < 0.0002; other orientations: *p* > 0.01; Bonferroni corrected). To understand the origin of the biases, we first derived the theoretical prediction of the distorted retinal image from the changes in optics (i.e., induced cylindrical diopters; Supplementary Fig. S2) and estimated the resultant orientation biases (Fig. 2C, dashed black line). The perceptual biases of the control group matched well with the prediction of the theoretical model (*t*_(9)_ = 1.171, *p* > 0.05). However, the perceptual biases of the chronic group were much smaller than the theoretical predictions (*t*_(46)_ = − 2.263, *p* < 0.05). This indicates that the reported perceptual biases of the control group mainly originated from the retinal distortion caused by astigmatism. The reduced perceptual bias in the chronic group suggests that long-term exposure to the astigmatic condition improved orientation perception, which cannot be explained by retinal input.

**Figure 3:**
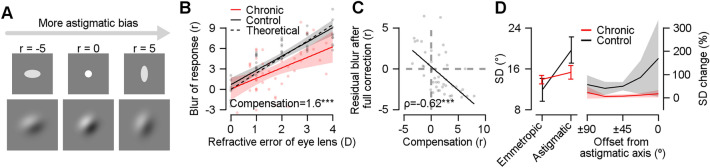
Automatic compensation of astigmatic distortion after chronic exposure. (**A**) The amount of optical blur (*r*) was estimated using the point spread function model. If the *r* value increases (as *r* = 5), a Gabor stimulus would be distorted to exhibit more astigmatic bias, and if the *r* value decreases (as *r* = − 5), it would be distorted to show the opposite bias, compared to emmetropia (*r* = 0). (**B**) The amount of optical blur (*r*) estimated in perceptual bias was plotted as a function of the physical refractive error of the eye in diopter (**D**). Dots and solid lines represent the *r* value of each eye and the linear regression line of each group. The shaded areas indicate ± 95% confidence intervals from bootstrapping. The amount of compensation indicates the mean vertical distance between the regression line of the control group (black line) and each red dot (***p < 0.001). (**C**) The relationship between the residual optical blur after fully correcting the refractive errors of the eyes and the amount of compensation of astigmatism. Rho indicates Spearman’s correlation coefficient (***p < 0.001). The solid line indicates the result of linear regression. (**D**) Standard deviations (SD) for each emmetropic and astigmatic vision condition and each group (left panel). The percent SD change in the astigmatic vision condition compared with the emmetropic condition for each orientation (right panel). The error bars and the shaded areas indicate ± 2 SEM.

Next, we used a point spread function model to quantify the amount of retinal distortion that corresponded to the perceptual biases in each chronic and control group (PSF; Fig. 3A). This approach has been widely used to simulate optical aberration^14,15^ and has successfully estimated the effective retinal distortion for each perceptual bias in a similar task^6^.

As with the bias comparison (Fig. 2C), the optical blur (retinal distortion) estimated from the perceptual errors (see Supplementary Fig. S3A for perceptual errors in each diopter group) were different across the groups (Fig. 3B; two-way ANOVA, *F*_*group* (1,275)_ = 21.140, *F*_*diopter* (4,275)_ = 21.787, *F*_*interaction* (4,275)_ = 3.612, *p*_s_ < 0.01). In the control group, the optical blur estimated from the perceptual errors linearly increased as the refractive errors of the optical organs increased, which closely followed the prediction of the theoretical model (overlapping black solid line and dashed line in Fig. 3B). However, most of the estimated optical blur in the chronic group was much lower than the prediction of linear regression calculated from the control group data (red dots below the solid black line in Fig. 3B; *t*_(46)_ = − 4.070, *p* < 0.001). This indicates that the extra-retinal process counteracts physical refractive errors when chronically exposed to astigmatism. Thus, we interpreted the reduction in optical blur in the chronic group as compensation.

### Automatic extra-retinal compensation counteracts the retinal distortion in chronic astigmatism

We further investigated whether the reduction of orientation bias originated from a fixed compensatory process that provides a constant compensation, or a variable compensatory process that adaptively changes the amount of compensation depending on the degree of optical blur. To test which hypothesis was correct, we fully corrected the eyes in the chronic group (emmetropia; Supplementary Fig. S1, upper right panel) and estimated the residual optical blur from the perceptual errors (Fig. 3C). Although all refractive errors were corrected (therefore, no retinal distortion was expected), we found that the participants made conspicuous, systematic perceptual errors. The residual optical blur estimated from the perceptual error was negatively correlated with the amount of compensation (ρ_45_ = − 0.62, *p* < 0.001). For those who showed less orientation perception error in the uncorrected astigmatism condition (compensation *r* > 0), there was an inverse bias in orientation perception when optical aberration was fully corrected (residual blur in full correction *r* < 0 in Fig. 3C; *t*_(34)_ = − 3.730, *p* < 0.001). This result suggests that compensation is an automatic extra-retinal process that provides constant compensation, regardless of the physical optical aberrations. In addition, the amount of compensation was constant, irrespective of the severity of astigmatism and the amount of correction. This was not correlated with the amount of cylindrical refractive error (Supplementary Fig. S3B; ρ_45_ = 0.23, *p* > 0.05), nor the amount of under-corrected cylindrical refractive errors in eyeglasses used daily (Supplementary Fig. S3C; ρ_45_ = − 0.12, *p* > 0.05). This strengthens the hypothesis that astigmatic vision is automatically compensated for.

The automatic extra-retinal process (which provides constant compensation) affects not only biases but also the variability of orientation perception. The variability in the control group with astigmatic vision was significantly higher than in the group with emmetropic vision. This is because inducing astigmatism using a cylindrical lens can cause optical image blur. However, if automatic compensation is provided in the chronic group, the increase in variability in astigmatic vision becomes weaker. This result was consistent with the prediction (Fig. 3D, left panel). There was a significant interaction between the effect of vision state and groups in standard deviations (SDs) of orientation perception (non-parametric two-way ANOVA^16^; *F*_(1,184)_ = 53.278, *p* < 0.01). In addition, the dependence of the variability in orientation perception on stimulus orientation was different between the chronic and control groups. In the control group, the relative SD of orientation perception in astigmatic vision over the SD in emmetropic vision linearly increased as the orientation of the Gabor approached the astigmatic axis (Fig. 3D, right panel, linear trend analysis, *F*_(4, 36)_ = 5.218, *p* < 0.05). This was predictable because the loss of orientation information increased and the resultant perception became noisier as stimulus orientation approached the astigmatic axis, consistent with previous studies^17^. However, the relative percent change in the chronic group did not depend on stimulus orientation (linear trend analysis, *F*_(4, 184)_ = 0.034, *p* > 0.05). Although the physical optical distortion of the retinal image predicted an identical variability increase as the stimulus orientation approached the astigmatic axis, the variability did not increase as a function of stimulus orientation. This indicates the automatic characteristics of the compensatory process that stabilizes the perception under chronic astigmatic vision.

### Adaptation caused by chronic astigmatism compensates for the optical distortion in orientation perception

Naturally, we suspected that the compensatory neural process added an inverse bias to the distorted orientation information from the retinal image. To understand the origins of the inverse bias, we extended the PSF model by adding neural modification of the retinal distortion as additional components. Thus, the extended model consisted of two types of bias: retinal distortion and neural modification.

**Figure 4:**
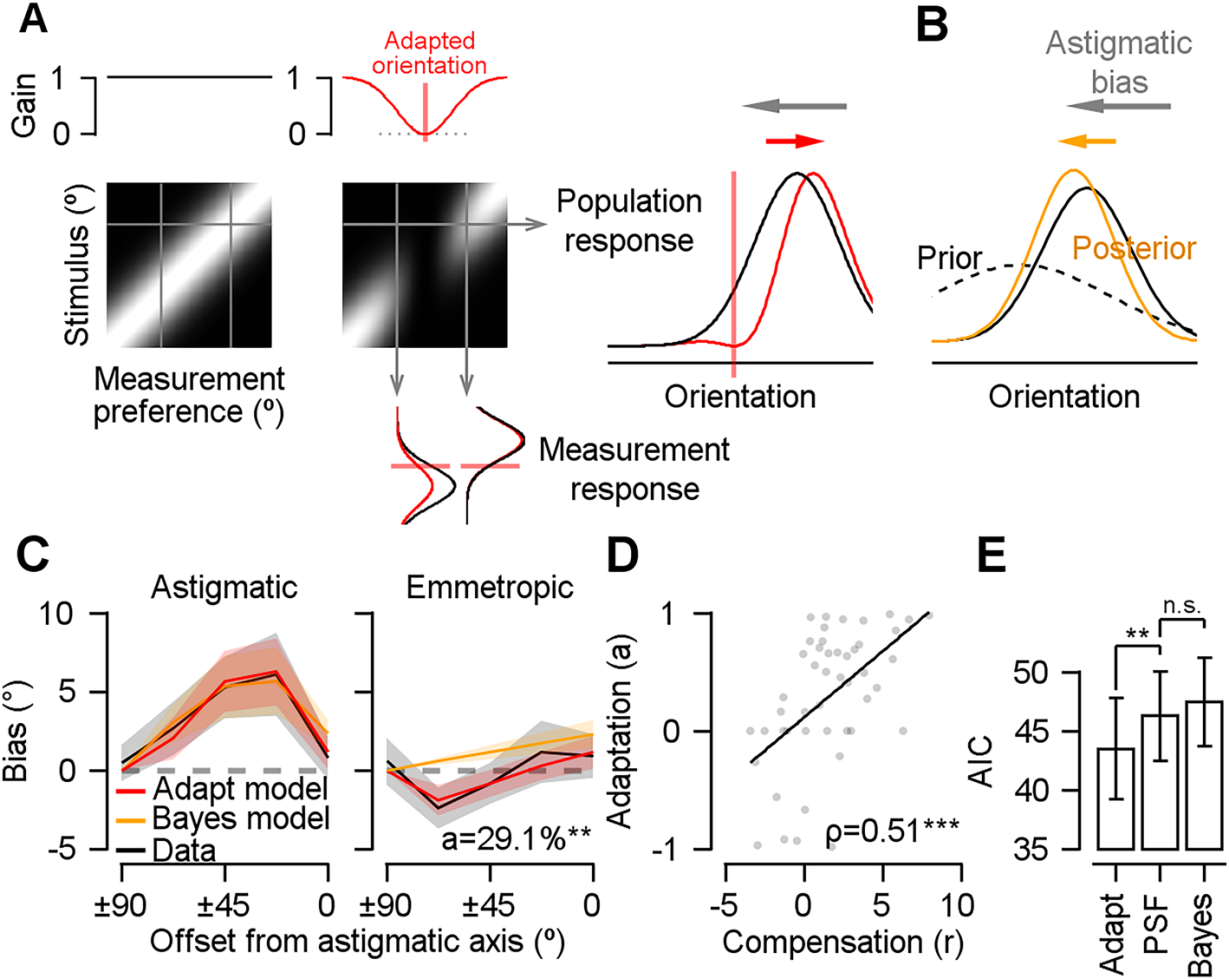
The result of the adaptation and the Bayesian model. (**A**) The response matrix of the adaptation model. The vertical axis represents the response of a single measurement to the various stimuli orientations, and the horizontal axis represents the population response to single stimulus orientation. The upper panels indicate each measurement’s gain function. A decrease in Gaussian shape gain around the adapted orientation results in the parametric reduction of each measurement’s response around the adapted orientation (lower panels). In population orientation tuning response, the reduction of gain around the adapted orientation causes the repulsive shift against the astigmatic bias (right panel). (**B**) In the Bayesian model, the prior expectation is built after long exposure to the biased distribution of astigmatism (dashed black line). Then, the outcome of perceptual inference, or posterior distribution (orange line), is shifted toward the prior expectation, conforming to the astigmatic bias. (**C**) Each model’s predictions for the perceptual bias in the astigmatic vision and the emmetropic vision condition (red: adaptation model; orange: Bayesian model) overlapped with the actual perceptual bias (black). The value *a* indicates the amount of gain–loss in percentage (**p < 0.01). The shaded areas indicate ± 2 SEM. (**D**) The relationship between the amount of gain loss and compensation in astigmatism. Rho indicates Spearman’s correlation coefficient (***p < 0.001). The solid line indicates the result of linear regression. (**E**) The corrected Akaike’s Information Criteria was estimated from the predictions of the adaptation model (adapt), point spread function model (PSF), and Bayesian model (Bayes) on the emmetropic vision state. The error bars indicate ± 2 SEM (**p < 0.01; n.s., *p* > 0.05).

One possible explanation for neural modification is orientation-specific neural adaptation. Eyes with astigmatism experience orientations orthogonal to the astigmatic axis more frequently than other orientations, due to optical distortion and the resulting retinal biases. This long-term adaptation could cause the visual system to lose sensitivity to selective orientations because of repeated activations (Fig. 4A, y-axis of the response matrix). As a result, neural population responses to stimulus orientations would be repelled away from the adapted orientation against the effect of astigmatic bias (Fig. 4A, x-axis of the response matrix, right panel). To estimate the amount of adaptation, we simulated the adaptation-induced neural response change around the adapted orientation with a model parameter that can control the gain of orientation-dependent responses.

The other possibility is that the visual system might develop a prior expectation for a stimulus orientation from the statistical distribution of stimuli: orientations orthogonal to the astigmatic axis would be experienced more frequently due to optical distortion (Fig. 4B). In this Bayesian inference model, the predicted outcome is the opposite of the prediction of the adaptation model. Following Bayes’ theorem, prior knowledge of the orientation distribution (prior probability) would attract the perception toward more frequent orientations (see Methods for details). The adaptation and Bayesian models were fitted to the perceptual errors obtained in both astigmatic and emmetropic vision states.

Both hypothetical models easily explained the perceptual errors in the astigmatic vision state (Fig. 4C, left panel), but only the adaptation model explained the inverse orientation bias in the emmetropic vision state (Fig. 4C, right panel). In both models, the parameters for retinal modification and neural modification can be adjusted to explain perceptual errors. Therefore, they can approximate the orientation bias in the astigmatic condition by adjusting the amount of retinal distortion (decreased r = − 0.64 ± 0.20 in Bayesian model and increased r = + 0.57 ± 0.55 in the adaptation model compared to the PSF model), but only the adaptation model can explain the bias in the emmetropic condition because there is no optical distortion and only parameters for neural modification can be adjusted. The Akaike information criteria showed that the adaptation model was better than the original PSF model in explaining the perceptual errors in the emmetropic vision states (Fig. 4E; *t*_(46)_ = − 2.986, *p* < 0.01), while the Bayesian model was not significantly different from the PSF model (*t*_(46)_ = 1.562, *p* > 0.05). The estimated gain parameters were significantly higher than zero, which indicates the occurrence of gain loss (gain–loss = 29.1 ± 16.2%, *t*_(46)_ = 3.523, *p* < 0.001). However, the estimated prior probability distribution in the Bayesian model was quite broad, indicating that it could not explain the observed pattern of perceptual errors (SD of the probability distribution = 139.2° ± 19.4°).

Moreover, the estimated gain loss in the adaptation model was highly correlated with the amount of compensation (Fig. 4D; ρ_45_ = 0.51, *p* < 0.001). That is, as more adaptation occurs around the orientation orthogonal to the astigmatic axis after chronic exposure to astigmatism, the greater the compensation for optical blur induced by astigmatic aberration. This correlation further supports that the reduction in the neural response gain to the orientation orthogonal to the astigmatic axis is an automatic process that benefits the visual system in reducing perceptual errors by adding repulsive biases. Furthermore, the inverse bias of the adaptation model was ± 67.5° away from the astigmatic axis. This is consistent with the fact that the difference between the chronic and control groups in the astigmatic vision states was prominent in a similar orientation range (Fig. 2C), suggesting that the adaptation model also explains the difference in the size of reduced biases across different orientations.

## Discussion

In the current study, we investigated the perceptual compensation in chronic astigmatic vision. We found that individuals with chronic astigmatism showed more accurate orientation judgments compared with individuals with normal vision in whom acute astigmatism was induced, even if the retinal input was systematically distorted. The enhancement in orientation perception was due to the automatic and stable compensatory extra-retinal process that counteracted the physical distortion of the retinal image. Additional modeling studies have suggested that automatic compensation might originate from orientation-specific adaptation, in which neural responses to orientations orthogonal to the astigmatic axis were suppressed.

Our results explain why edges and lines of images appear evenly distributed and continuously aligned, even under minor astigmatism in daily life (Fig. 1). Owing to optical aberrations of astigmatic vision, the majority of edges in the retinal image are biased toward certain orientations; thus, the objects’ natural contours should be lost. However, after prolonged exposure to the systematic distortion of retinal images, the compensatory process fills in the missing edges that are orthogonal to the biased distribution of orientations, and partly restores them. It also emphasizes that our visual system actively modifies and modulates incoming sensory information for stable visual perception. Therefore, both the distortion in the retinal image and the potential neural compensation should be considered when any optical aberration occurs.

Our findings on the automatic compensatory process link everyday astigmatic visual perception with neural adaptation. Adaptation has been suggested as a possible mechanism for the perceptual compensation of optical aberrations. A growing number of studies have demonstrated that transient adaptation to optical blur improves the quality of impaired vision^8,9,18–20^. Thus, neural adaptation after long-term exposure may play a key role in compensating for optical blur^10,21,22^. However, previous studies only reported empirical observations without providing mechanistic neural mechanisms of how neural adaptation enhances perception, partly because they did not have systematic experimental conditions that could constrain the neural models. Extending from previous studies, we measured orientation perception directly by utilizing the Gabor stimulus, which is known to resemble the shape of orientation-selective cells’ receptive fields in the primary visual cortex. Measuring orientation perception allowed us to clarify the link between compensation and adaptation. Based on orientation-dependent neural adaptation of cells, the population orientation tuning curves are repulsively shifted against the retinal astigmatic bias, resulting in constant automatic compensation in orientation perception.

Future research may expand our findings in several ways. The automatic property of the compensatory process can explain why some eyeglass wearers feel uncomfortable when their astigmatic errors in optical organs are fully corrected^7^. The compensatory process would reduce the perceptual errors in the presence of astigmatic defocus but cause counteracting perceptual distortion when the physical optical distortion is fully corrected. It would also be interesting to see how the compensatory process interacts with other optical phenomena, as astigmatic vision is closely associated with various optical features^23–25^. Distinctive temporal stages during astigmatic perception^6^ may be useful for elucidating interactions with other optical phenomena. Another intriguing and practical question is to determine the relationship between the amount of refractive error and compensation in a specific range of optical aberrations in astigmatism. A positive correlation between them was reported for astigmatic aberrations lower than 1.50 D^10^. We did not observe these relationships, probably because of the much larger scale of optical aberrations used in the current study (Supplementary Fig. S3B, ranging from 0.25 to 4.00 D). It would be of clinical importance to specify the amount of compensation for various sizes of optical aberration when deciding on the proper correction^26^.

There is much more to be revealed about the neural mechanisms of compensation. The detailed mechanisms and substrates of adaptation are known to depend on the duration of adaptation^27,28^. Thus, it is worth questioning whether the neural compensatory mechanisms in astigmatism also depend on the duration of experience with astigmatism, from a few seconds, to hours, to years. These findings have several clinical implications. Although behavioral performance may be similar^10^, astigmatism may not be corrected in the same manner if the neural mechanisms are fundamentally different depending on the amount of exposure to astigmatic vision. It may be optimal for ophthalmologists to diagnose with sufficient intervals, consider different neural compensations for astigmatism, and correct the refractive errors through adaptive optics^29,30^ to minimize dizziness after correction.

The neural mechanisms underlying astigmatism across brain regions must also be elucidated. We do not yet know whether the entire compensation mechanism originates from a purely sensory process or from higher-level cognitive factors. However, at least the constant and automatic components appear to originate from the modulation in the sensory processes, as our neural adaptation model supports. Indeed, studies have shown that impairment of optical blur can be mitigated in sensory-related neural substrates, such as optical nerves^31^, in a brain region related to binocular vision^32^, or in the early visual cortex^33^. However, these results do not exclude the involvement of higher hierarchical information processes in astigmatic compensation. Instead, they suggest that the workings of higher cognitive functions may be expressed in the responses of lower visual cortical neurons after long-term exposure to astigmatism. Therefore, different brain regions, ranging from the early visual cortex to the higher-order cortical regions, may be involved in determining the various characteristics of compensation in chronic astigmatism. Future studies may reveal more details on how different brain regions contribute to the dynamics of astigmatic perception.

## Methods

### Two groups of participants: chronic astigmatism and normal-vision control group

Behavioral data were collected from 37 participants (23 men and 14 women; mean age, 21.3 ± 3.2). Patients who underwent eye surgery within the last six months were excluded from the experiment. Before the experiment, refractive errors of the naked eye were evaluated using an auto refractometer (AR; Huvitz, HRK-7000, Republic of Korea). We measured the refractive errors using an AR to minimize the unwanted variability that could occur if measured manually. Participants were then divided into two groups: a chronic astigmatism group and a normal vision control group. Participants were classified into the control group if they had 20/20 vision or better with their naked eyes. Data were collected only from participants’ dominant eyes. This criterion was used because the participants with 20/20 vision or better naturally had a very small amount of refractive errors (see table 20-1 in ref.^34^). We identified the dominant eye based on monocular preference in binocular viewing^35^. If the participants had vision worse than 20/20, they were assigned to the chronic group, and data were obtained from each using monocular vision. We discarded data from four eyes with high cylindrical refractive powers (over 4.00 D) and from three eyes with high under-correction in their daily eyeglasses (under − 1.00 D) because of the small sample size. As a result, datasets of 47 eyes in the chronic group (27 participants) and 10 datasets in the control group (10 participants) were used for further analysis. Additionally, the refractive errors of daily eyeglasses (or contact lenses) were documented to identify their usual under-corrected refractive errors. The measured refractive errors of the naked eye and with eyeglasses of the chronic group are presented in Table 1. It specifies the spherical and cylindrical refractive errors and the axes of the cylindrical refractive errors of each eye. The Institutional Review Board of Sungkyunkwan University approved the study (IRB 2018-05-003), and all studies were performed in accordance with the relevant guidelines. All participants provided written informed consent and were blinded to the purpose of the experiment.

### Experimental conditioning

The experiments consisted of two vision conditions (emmetropic or astigmatic) for each group of participants. In the chronic group with emmetropic vision condition, to ensure that the light rays evenly focused on the retina across visual space, we placed trial lenses in front of the participants’ eyes so that they fully corrected both the spherical and cylindrical refractive errors measured with the AR (Supplementary Fig. S1, upper right panel). In the control group, we only placed a Plano lens for fair comparison because their vision was already near-emmetropic (Supplementary Fig. S1, lower right panel).

In the astigmatic vision condition, the light rays in a certain meridian were focused before the retina (myopic regular astigmatism, as shown in Fig. 2A). In the chronic group, the spherical refractive errors were fully corrected, leaving cylindrical refractive errors unchanged (Supplementary Fig. S1, upper left panel). As a result, the vision of the chronic group was dominated by cylindrical refractive errors. To induce a compatible astigmatic state in the control group, we used a cylindrical lens varying from + 1.00 to + 4.00 D, with a step size of + 1.00, with the astigmatic axis set horizontally (Supplementary Fig. S1, lower left panel).

We added a + 1.50 D spherical lens in front of the test eye to allow light rays to penetrate the lens parallel in both groups (the first rows in each panel in Supplementary Fig. S1). We also corrected minor residual spherical errors that might remain in the control group (20/20 vision) using the fogging method^34^. We first blurred the vision by placing an additional + 1.50 D spherical lens (fogging), and then reduced the power with a step size of 0.25 D until the participants could read the Snellen chart (defogging). In the contralateral eye, we placed a + 10.00 D spherical lens to prevent intervention to the test eye. The participants were instructed to keep both sides of their eyes open.

### Stimuli and task design

Participants were asked to report the perceived mean orientation of a tilted Gabor array presented on the monitor at a viewing distance of 60 cm (Fig. 2B). We used a gamma-corrected, 20-inch CRT monitor with a spatial resolution of 800 × 600 pixels and a vertical refresh rate of 85 Hz (Hewlett Packard p1230; maximum and minimum luminance of 106.0 cd/m^2^ and 0.8 cd/m^2^, and gray background luminance 55.8 cd/m^2^). The Psychophysics Toolbox for MATLAB (Mathworks Inc.)^36,37^ was used to control the presentation of all stimuli. The experiments were conducted in a dark room.

The participants initiated each trial by pressing a space bar. After 400 ms of a blank screen, they were presented with 20 Gabor stimuli (0.25° radius) for 153 ms within an invisible boundary (2.5° radius). Each Gabor was randomly positioned from trial to trial and had a spatial frequency of 2 cycles/° with the Michelson luminance contrast set to 60%. We used a sinusoidal Gabor patch with randomized polarity (phase of 0° or 180°). The orientations of all Gabor stimuli were the same in a given trial and randomly chosen from eight orientation deviations from the astigmatic axis of the test eye (0°, ± 22.5°, ± 45°, ± 67.5°, or 90°). After 494 ms of the post-stimulus blank screen, a randomly tilted white bar (5° radius) appeared at the center of the screen. The participants rotated the orientation bar to the perceived orientation of the array by moving the mouse and clicking the left mouse button. The participants were asked to fixate on a white fixation point (radius of 0.1°) during the trial.

The participants in the chronic group performed eight blocks of trials (two blocks for each vision condition and each eye). The participants in the control group performed 10 blocks of trials (2 blocks for each possible cylindrical diopter condition: Plano, + 1.00, + 2.00, + 3.00, and + 4.00). Each block consisted of 120 trials, and the order of the blocks was randomized. The participants were not informed of the block conditions or their performances. The total duration of the experiment was 1 to 1.5 h, and the participants wore a specific lens for 15 min on average. We controlled the duration of the experiment to minimize unwanted visual acuity improvements induced by adaptation to the optical settings^10,20^ in the control group.

### Estimation of response bias and variability

In each eye and condition, we quantified the distortion of orientation perception in the following steps using a circular statistics toolbox^38^. First, perceptual errors were obtained from the difference between the stimulus orientation and the reported orientation. To reduce individuals’ clockwise or counterclockwise biases in the errors, we subtracted the average error of all trials from individual errors. Then, we changed the signs of the errors when stimulus orientations were between 0° and 90°, and the data were merged according to the offset from the astigmatic axis (e.g., trials showed + 45° tilted from the astigmatic axis and − 45° tilted trials were collapsed). Finally, astigmatic biases were obtained by averaging the response errors for each orientation offset from the astigmatic axis (Fig. 2C and Supplementary Fig. S3A). The positive value of the astigmatic bias indicated that the response was biased away from the astigmatic axis, and a negative value indicated that the response was biased toward the astigmatic axis.

We also measured the response variability with the SD of the behavioral errors. The SDs in each condition and group were computed for all orientations combined (Fig. 3D, left panel) or for each orientation. To estimate the relative variability difference between astigmatic and emmetropic vision conditions, we calculated the percent change of SD in the astigmatic condition from the SD in the emmetropic condition (Fig. 3D, right panel).

### Point spread function (PSF) model

Next, we estimated the effect of the optical blur on the response bias pattern. Specifically, we estimated the amount of blur in the retinal image that caused a response bias in each orientation using the simple PSF model^6^. The model mimics the optical property of the astigmatic vision that a light ray spreads elliptically using an oval-shaped convolution kernel (*K*) (Fig. 2A compares the shape of a light ray before and after lens). We used a single parameter (*r*) that adjusted the axis of the kernel with an elliptical shape.

1 $$\begin{aligned} & K_{{\left( {x,y} \right)}} = \left\{ {\begin{array}{*{20}l} {\frac{1}{\pi \cdot v \cdot h},} \hfill & { \left( \frac{x}{h} \right)^{2} + \left( \frac{y}{v} \right)^{2} < 1} \hfill \\ {0,} \hfill & {else} \hfill \\ \end{array} } \right. \\ & \quad \left( {where \left( {v,h} \right) = \left\{ {\begin{array}{*{20}c} {\left( {1,\left| r \right| + 1} \right), if r \geqq 0} \\ {\left( {\left| r \right| + 1,1} \right), if r < 0} \\ \end{array} } \right.} \right) \\ \end{aligned}$$

A positive *r* value indicates that each light ray is scattered orthogonally to the astigmatic axis, zero indicates an emmetropic state, and a negative *r* value indicates that light rays are scattered along the astigmatic axis (Fig. 3A). As the size of the parameter *r* increases, the optical blur that induces astigmatic vision becomes more severe. To simulate the retinal image, we convolved the kernel with an image of oriented Gabor stimuli (eight possible orientations). Finally, the orientation was decoded by detecting the pixel boundaries that changed color from white to black at the center of the convolved Gabor stimulus. This orientation extraction resembles the orientation computation of simple cells in the primary visual cortex, optimized for edge detection^39^. To obtain the orientation errors from the simulation, we obtained the difference between the corresponding stimulus orientation and decoded orientation. Parameter *r* is the only free parameter in the PSF model. The parameter was estimated by finding the best-fitted model that minimized the sum of squared errors between the perceptual orientation error and the error calculated from the model, using the simulated annealing algorithm in the global optimization toolbox in MATLAB. The model was fitted to each eye’s data and visual conditions.

Using the PSF model, we compared the sizes of perceptual compensation in astigmatism between the chronic and control groups (Fig. 3B,C). First, we measured the relationship between the estimated parameter *r* and the optical refractive error measured using an AR machine. We fitted a simple linear regression line to the relationship between *r* and the optical refractive error in the astigmatic vision state. The average of the regression line was computed after bootstrapping 1,000 times (solid lines in Fig. 3B). We then computed the compensation by comparing each parameter *r* of the chronic group individuals on a given refractive error with the *r*-value from the control group’s average regression line on the refractive error (i.e., the vertical distance between a solid black line and each red dot). The positive compensation indicates that the parameter *r* estimated from the chronic group was smaller than the r-value of the linear regression line estimated from the control group (Fig. 3C).

### Theoretical model

We calculated the theoretical prediction of parameter *r* derived from the given optical aberration (Fig. 3B, dashed black line).

2 $$\begin{aligned} & r_{theoretical} = \frac{B}{P} - 1 \\ & \;\;\left( {where\;B = L \cdot \frac{{N - N_{astig} }}{{N_{astig} }}, N_{astig} = \frac{1}{{\frac{1}{{N_{emme} }} + \alpha }}, N_{emme} = \frac{L \cdot N}{{P + L}}, P = N \cdot \frac{S}{V}} \right) \\ \end{aligned}$$

B and P indicate the length of the retinal image, orthogonal to the astigmatic axis, of the astigmatic and emmetropic eyes respectively (see Supplementary Fig. S2 for graphical illustration). L, N, N_astig_, and N_emme_ denote pupil size, distance from the nodal point to the retina, and distance from the nodal point to the focal point in the astigmatic or emmetropic eye. We assumed a nodal distance of 24 mm^40^ and a pupil size of 2 mm when facing the bright screen, which is the typical size of adult eyes. α is the induced astigmatism in the diopter (from Plano to 4.00 D). S and V are the actual pixel size of the screen (≈ 0.50 mm) and viewing distance (60 cm), respectively. Using r_theoretical_, we generated a blurred retinal image and estimated the orientation biases in an identical manner to the PSF model (Fig. 2C, dashed black line).

### Adaptation and Bayesian model

We considered two hypothetical neural modifications that could adjust our orientation perception in astigmatic vision: adaptation and Bayesian models. We assumed that the neural modifications and retinal distortions caused by astigmatic vision would be additive. Thus, we added errors computed from the two neural models to the retinal errors predicted from the PSF model described above. The following description shows how the errors of each neural modification are computed. In the adaptation model, we first created a two-dimensional response matrix, wherein each column vector represented the response distribution to the stimuli of a single measurement (Fig. 4A). For simplicity, the response distribution is assumed to be normal. Then, the measurement response was multiplied by the gain value to obtain the gain-modulated response matrix (Fig. 4A, right response matrix). These gain values decreased following a normal distribution whose mean was set to be the orientation orthogonal to the astigmatic axis, and the SD was three times greater than the SD of the measurement’s response. A similar model with gain modulation effectively simulated neural adaptation in the primary visual cortex^41^. Finally, the population response of the measurements was obtained from the row vector of the response matrix. The population response was normalized and transformed into a probability distribution function. We used the average of the probability distribution as a model prediction of perceived orientation. The free parameter *a* adjusts the amplitude of the gain value function, a positive value of *a* indicates a reduction in gain at the adapted orientation (as Fig. 4A where *a* was 1), zero means no change in gain function regardless of orientation (as Fig. 4A where *a* was 0), and a negative value indicates an increase in the gain near the adapted orientation.

In the Bayesian model, we assumed that the observer would build up a certain expectation after affluently experiencing biases toward the orientation orthogonal to the astigmatic axis (Fig. 4B). Thus, we built a simple Bayesian observer model in which the orientation estimate would be the result of the reliability-based weighted sum of the likelihood function and the prior probability distribution if both followed a Gaussian distribution^42^.

3 $$\mu_{percept} { } = \frac{{\frac{{\mu_{lik} }}{{\sigma_{lik}^{2} }} + \frac{{\mu_{pri} }}{{\sigma_{pri}^{2} }}}}{{\begin{array}{*{20}c} {\frac{1}{{\sigma_{lik}^{2} }} + \frac{1}{{\sigma_{pri}^{2} }}} \\ \end{array} }}$$

$\mu_{lik}$ and $\sigma_{lik}$ denote the mean and SD of the likelihood function. $\mu_{pri}$ and $\sigma_{pri}$ denote the mean and SD of prior distribution, respectively. $\mu_{lik}$ was the given stimulus orientation, and ${\mu }_{pri}$ was set to the orientation orthogonal to the astigmatic axis. ${\sigma }_{pri}$ was a free parameter in the model.

Both the adaptation and Bayesian models were fitted as follows. First, the difference between each model’s mean estimate and stimulus orientation was computed. There were eight possible orientations for each vision state, so this yielded 16 error values. Second, the corresponding 16 astigmatic errors were computed using the PSF model described above. Then, the errors of the preceding two steps were summed for each corresponding orientation to obtain the predicted perceptual errors of the model. Although the SDs of the likelihood function of both models were not equal to the SDs of the resulting response distributions, we assumed they were the same for the sake of model simplicity, as this assumption is not a key factor for the pattern of the error. Indeed, the general shape of the model predictions was identical regardless of whether we set the SD of the likelihood function as an additional free parameter or used the SD of the behavioral distribution for model simplicity (Supplementary Fig. S4). Therefore, we estimated two free parameters on each model (*r* and *a* for the adaptation model; *r* and ${\sigma }_{pri}$ for the Bayesian model) to minimize the sum of squared error between the perceptual orientation errors and estimated orientation errors from each model (Fig. 4C).

To quantitatively evaluate which of the three models (PSF, adaptation, and Bayesian model) explained the behavioral response errors of the emmetropic condition better, the corrected Akaike’s Information Criteria (AICc)^43^ were used (Fig. 4E).

4 $$AIC_{c} = Nln\left( \frac{SS}{N} \right) + 2K + \frac{{2K\left( {K + 1} \right)}}{N - K - 1}$$

where N, K, and SS denote the number of data points (i.e., 16; eight orientations per condition), number of free parameters plus one, and the sum of squared errors, respectively.

## Supplementary Information

Supplementary Figures.

## Data Availability

The datasets generated and/or analyzed during the current study are available from the corresponding author upon reasonable request.
