# Supplementary material for: Automatic compensation enhances the orientation perception in chronic astigmatism
Source: Sci Rep. 2022 Mar 8;12:3710. doi: 10.1038/s41598-022-07788-y (PMC8904485; doi:10.1038/s41598-022-07788-y)
Supplement: Supplementary file 1 — Supplementary Figures. [file 41598_2022_7788_MOESM1_ESM.pdf]

Automatic compensation enhances the orientation perception in chronic astigmatism

Sangkyu Son[1,2], Won Mok Shim[1,2,4], Hyungoo Kang[*3], and Joonyeol Lee[*1,2,4]

[1]Center for Neuroscience Imaging Research, Institute for Basic Science (IBS), Suwon 16419, Republic of Korea

[2]Department of Biomedical Engineering, Sungkyunkwan University, Suwon 16419, Republic of Korea

[3]Department of Optometry, Catholic Kwandong University, Gangneung 25601, Republic of Korea

[4]Department of Intelligent Precision Healthcare Convergence, Sungkyunkwan University, Suwon 16419, Republic of Korea

[*]Proofs and correspondence to:

Joonyeol Lee
Department of Biomedical Engineering
Sungkyunkwan University
2066 Seobu-ro, Jangan-gu, Suwon-si
Gyeonggi-do, 16419, Republic of Korea
Tel: +82 (31) 299-4359
Email: joonyeol@g.skku.edu

OR

Hyungoo Kang
Department of Optometry
Catholic Kwandong University
24, Beomil-ro 579beon-gil, Gangneung-si,
Gangwon-do, 25601, Republic of Korea
Tel: +82 (33) 649-7375
Email: hgkang@cku.ac.kr

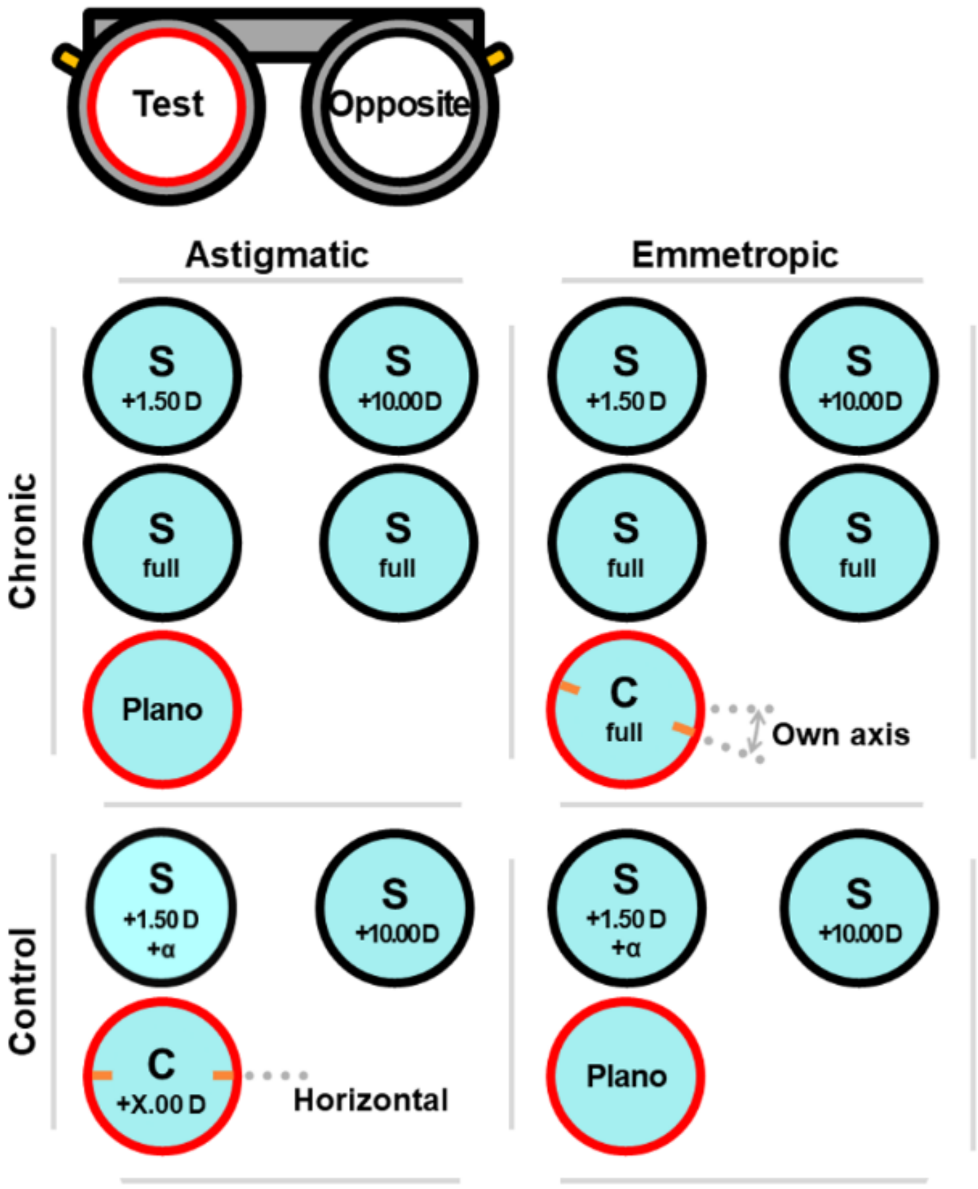

Supplementary Figure S1.

Experimental conditioning of each eye. Each panel indicates the lens used in the chronic (upper row) or control group (lower row) participants in the astigmatic (left column) or emmetropic vision conditions (right column). The notations S, C, full, α, and X indicate a spherical lens, cylindrical lens, full correction, additional spherical lens determined by the fogging method, and diopter of a cylindrical lens ranging from +1.00 to +4.00, respectively. The lenses with critical manipulation of each condition are marked with a red boundary. In the astigmatic vision condition, a Plano lens was placed in front of the test eye of the chronic group so that the participant would perform the task under one's own astigmatism (upper left panel), but a cylindrical lens was used instead in the control group to induce astigmatism (lower left panel). In the emmetropic condition, we placed the cylindrical lens with its own axis to the chronic group participants to fully correct the refractive errors (upper right panel). The control group participants wore a Plano lens to perform the task with their own vision (lower right panel).

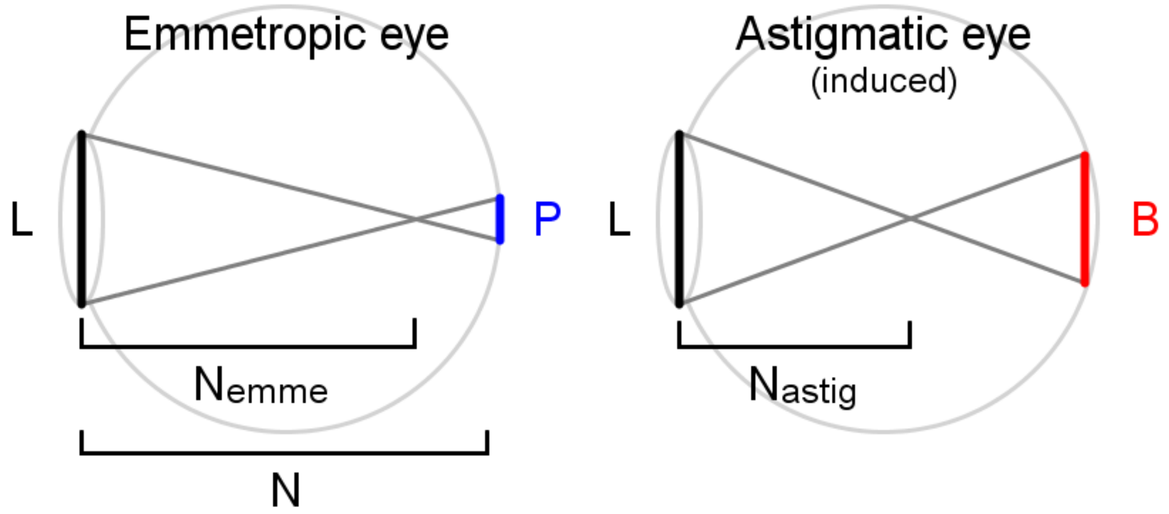

Supplementary Figure S2.

Illustration of the veridical model in the orthogonal plane to the astigmatic axis. (Left) In the case of the emmetropic eye, the light ray passes through the pupil (size of L) to form a retinal image (length of P). Since P is small enough, the distance from the nodal point to the focus (N_{emme}) approximates the distance from the nodal point to the retina (N). (Right) In the case of the astigmatic eye, the light ray is more refracted and focuses in front of the retina, compared with the focus of the emmetropic eye ($N_{emme} > N_{astig}$). This causes the retinal image to be blurred (lengthened) as B.

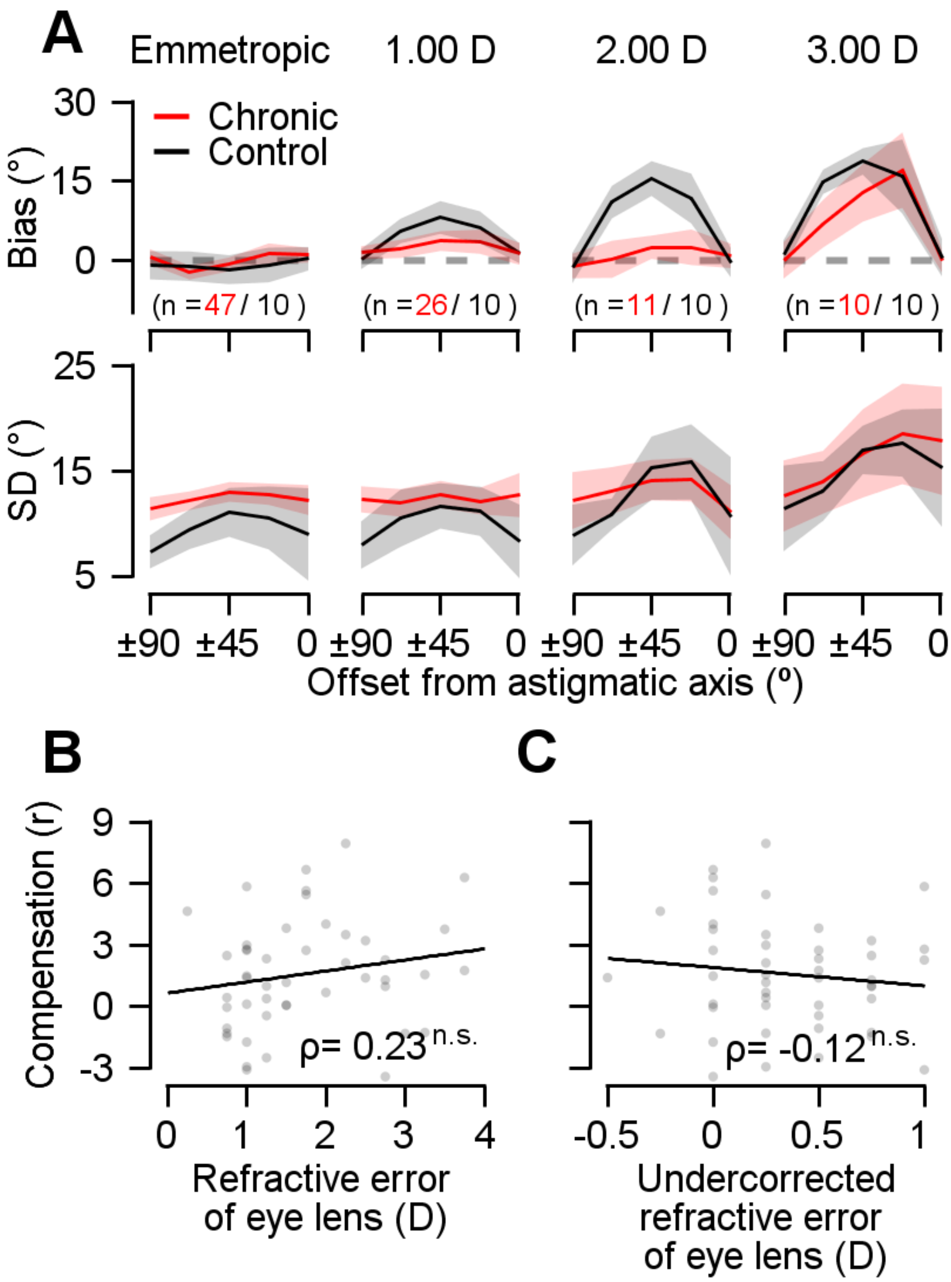

Supplementary Figure S3.

A. The means and standard deviations (SDs) of perceptual errors for each diopter of the cylindrical refractive error. Each participant was assigned to the nearest diopter out of three diopters. The number of participants of each bin for chronic and control groups is shown at the bottom of the figure. The shaded areas indicate ±2 SEM. **B and C.** The relationship between the size of perceptual compensation estimated from participants with chronic astigmatism and the size of cylindrical refractive errors measured from their eye lens (B) or the size of uncorrected cylindrical refractive errors in their daily use eyeglasses (C). Rho indicates Spearman's correlation coefficient (n.s., $p > 0.05$). The solid line indicates the result of the linear regression.

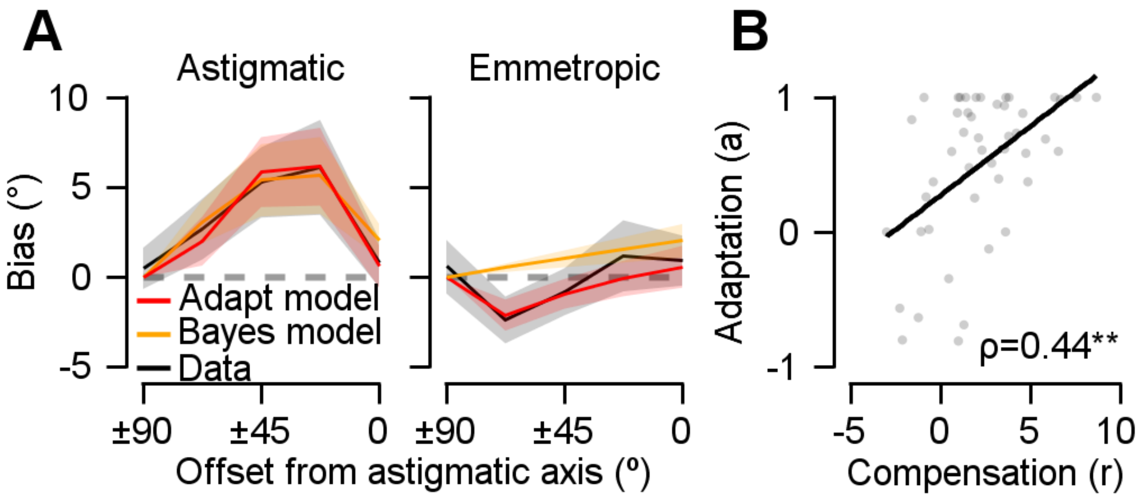

Supplementary Figure S4.

The results of the adaptation and Bayesian model, without assuming the standard deviations of the likelihood function being equal to the standard deviations of the response distributions. The model predictions of the perceptual bias in each vision condition (A) and the relationship between the amount of gain loss and compensation (B) are shown. The shaded areas, rho, and the solid line indicate ±2 SEM, Spearman's correlation coefficient (**, $p < 0.01$), and the result of linear regression, respectively.
